# Advances in the Biology of Seed and Vegetative Storage Proteins Based on Two-Dimensional Electrophoresis Coupled to Mass Spectrometry

**DOI:** 10.3390/molecules23102462

**Published:** 2018-09-26

**Authors:** Daniel Mouzo, Javier Bernal, María López-Pedrouso, Daniel Franco, Carlos Zapata

**Affiliations:** 1 Department of Zoology, Genetics and Physical Anthropology, University of Santiago de Compostela, 15782 Santiago de Compostela, Spain; daniel.mouzo.calzadilla@usc.es (D.M.); javier.bernal.pampin@gmail.com (J.B.); mariadolores.lopez@usc.es (M.L.-P.); 2 Meat Technology Center of Galicia, 32900 San Cibrao das Viñas, Ourense, Spain; danielfranco@ceteca.net

**Keywords:** seed proteomics, seed phosphoproteomics, seed glycoproteomics, seed quality traits, seed molecular breeding

## Abstract

Seed storage proteins play a fundamental role in plant reproduction and human nutrition. They accumulate during seed development as reserve material for germination and seedling growth and are a major source of dietary protein for human consumption. Storage proteins encompass multiple isoforms encoded by multi-gene families that undergo abundant glycosylations and phosphorylations. Two-dimensional electrophoresis (2-DE) is a proteomic tool especially suitable for the characterization of storage proteins because of their peculiar characteristics. In particular, storage proteins are soluble multimeric proteins highly represented in the seed proteome that contain polypeptides of molecular mass between 10 and 130 kDa. In addition, high-resolution profiles can be achieved by applying targeted 2-DE protocols. 2-DE coupled with mass spectrometry (MS) has traditionally been the methodology of choice in numerous studies on the biology of storage proteins in a wide diversity of plants. 2-DE-based reference maps have decisively contributed to the current state of our knowledge about storage proteins in multiple key aspects, including identification of isoforms and quantification of their relative abundance, identification of phosphorylated isoforms and assessment of their phosphorylation status, and dynamic changes of isoforms during seed development and germination both qualitatively and quantitatively. These advances have translated into relevant information about meaningful traits in seed breeding such as protein quality, longevity, gluten and allergen content, stress response and antifungal, antibacterial, and insect susceptibility. This review addresses progress on the biology of storage proteins and application areas in seed breeding using 2-DE-based maps.

## 1. Introduction

Storage proteins accumulate during seed development within membrane-bound organelles called protein bodies and serve as a reservoir of amino acids, reduced nitrogen, carbon, and sulfur required for germinating seedlings [1,2,3,4,5]. Storage proteins also play a crucial role in human nutrition and livestock feed. Plants provide most (ca. 58%) of the dietary protein consumed worldwide compared to animal-based protein sources, although with marked variations depending on the region and economic status [6,7,8,9]. In particular, seeds are a major source of the dietary protein content that varies approximately from 10% (dry weight) in cereals to 40% in some legumes and oilseeds [1]. Storage proteins determine to a great extent the seed nutritional quality because they account for a major part of the total protein content. By way of illustration, approximately 70–80% of the total amount of reduced nitrogen in cereals and legume grains can be attributed to seed storage proteins (SSPs) [10]. In addition, some SSPs and vegetative storage proteins (VSPs) can exhibit additional enzymatic activities such as lipid acyl hydrolase, acyltranferase, esterase and acid phosphatase activities capable of assuming useful supplementary biological functions, including defense and antioxidant functions [11,12,13,14].

The model species *Arabidopsis thaliana* L. has played a key role in identifying gene regulatory networks that govern seed development and germination. A wide repertoire of genetic technologies enabled the identification of essential regulatory genes during seed development and germination in Arabidopsis as well as the identification of orthologous genes in other plant species [15,16,17,18,19,20]. These technologies include forward genetic screens of lines obtained by T-DNA insertional mutagenesis for tagged mutants that produce a knockout phenotype, microarray RNA transcriptional profiling, and identification of seed-specific transcription factors (TFs). Genes involved in the regulatory networks responsible for the synthesis, accumulation and mobilization of seed storage proteins have been identified in Arabidopsis and other plants [20,21]. Dormancy induction and germination are greatly regulated by the dynamic balance between the functional antagonist abscisic acid (ABA) and gibberelic acid (GA) phytohormones [22]. Considerable progress has been achieved in unraveling the regulatory mechanisms underlying ABA response [23,24,25,26]. In particular, a number of protein-coding genes and TFs have been associated with the hormonal regulation involved in the synthesis and accumulation of storage proteins [20].

Seed proteome comprises a heterogeneous collection of functionally differentiated proteins that undergo highly dynamic qualitative and quantitative changes in order to meet seed requirements during development and germination. Storage proteins are typically multimeric proteins encoded by multi-gene families constituted by highly homologous genes clustered on one or various chromosomes [14,20,27,28]. They often undergo abundant glycosylations and phosphorylations, two types of co- and/or post-translational modifications (PTMs) that notably increase the diversity of isoforms [29,30]. Proteomics encompasses a wide range of technologies with sufficient potential for the detailed characterization of the broad set of storage protein isoforms. There have been a large number of gel-based and gel-free MS-driven proteomic studies focused on seed proteome [31,32,33,34,35,36,37]. The 2-DE proteomic technology initially developed by O’Farrel [38] opened the way to numerous studies addressing the characterization of storage proteins. Reference maps of many storage proteins have been constructed based on the separation of total seed proteins by 2-DE and protein identification by downstream MS analysis.

2-DE-based maps of storage proteins have been obtained using two different experimental strategies with strengths and weaknesses. Hundreds of publications have used experimental protocols for the study of global seed proteins with very different relative abundance [31,32,33,34,35,39,40,41]. This is an optimal experimental approach to assess the interplay between storage proteins and other seed proteins, but it entails the loss of definition of storage protein isoforms on 2-DE gels. Alternatively, a minority of studies used 2-DE specific protocols aimed at obtaining high-resolution profiles of storage proteins [29,30,42,43,44]. This approach is very useful to characterize storage protein isoforms and their response to internal and external seed stimuli at higher level of resolution, although the information it provides is decoupled from the rest of seed proteins. Overall, the application of these two strategies has provided most of the advances in the biology of storage proteins. These advances cover facets as diverse as the identification of isoforms and their relative abundance, the identification, mapping and quantitation of phosphorylated and glycosylated isoforms and the assessment of qualitative and quantitative changes of isoforms during seed development and germination. Seed breeding programs have benefited from these advances for the improvement of many seed traits of interest such as protein quality, longevity, gluten and allergen content, stress response and antifungal, antibacterial and insect susceptibility [45,46,47,48,49,50,51].

This review focuses on the use and importance of 2-DE-based maps to obtain insights into the biology of storage proteins and application areas in seed breeding.

## 2. Terminology and Classification of Storage Proteins

SSPs are currently denominated according to profundly heterogeneous criteria: extraction/solubility in distinct solvents (e.g., albumins), sedimentation coefficients (e.g., 7S), generic names in Latin (e.g., hordeins from barley, *Hordeum vulgare* L.), trivial names (cactin from *Cereus jamacaru* DC.) and specific terminology for polypeptide subunits encoded by multigene families (e.g., phaseolin α-type polypeptide from common bean, *Phaseolus vulgaris* L.) [34]. However, most storage proteins have traditionally been classified into four main groups on the basis of their solubility in different solvents as proposed by Osborne [52]: water (albumins), dilute saline (globulins), alcohol-water mixtures (prolamines) and dilute acid or alkali (glutelins). New bioinformatics algorithms have recently been proposed for a higher classification accuracy using specific sequences available in public databases [53,54].

VSPs are a differentiated set of plant storage proteins located in vegetative tissues (tubers, stems, roots or leaves) of plants such as the sweet potato (sporamins), the potato (patatins) and *Oxalis tuberosa* Mol. (ocatins) [2,13,55,56]. For example, the patatin multigene family can be divided into class-I and class-II gene subfamilies with differential tissue expression patterns: class I transcripts are potato (*Solanum tuberosum* L.) tuber specific while class II transcripts are expressed not only in tubers but also in roots but much less abundant than class I transcripts [57,58]. VSPs are not grouped together with SSPs because they belong to a family of unrelated proteins and exhibit certain different characteristics such as a distinct form of mobilization [2,13,55,59].

A representative list of storage proteins (SSPs and VSPs) that includes important worldwide agricultural crops is shown in Table 1. 

Globulins predominate in dicotyledoneous seeds whereas prolamins are the major storage proteins in most cereals. Globulins are located in the embryo and outer aleurone layer of the endosperm and are commonly divided according to their different sedimentation coefficients (7S and 11S). They are very similar to 7S vicilins in legumes and other dicotyledoneous plants [3]. In maize (*Zea mays* L.), globulins are classified as globulin-1, the most abundant storage protein in embryos, and globulin-2. In soybean (*Glycine max* L.), the seeds contain a considerable amount of globulins, namely β-conglycin (7S globulin) and glycinin (11S globulin). β-conglycin has a trimeric structure composed of α, α′, and β subunits with molecular weights ranging from 50 to 76 kDa. Glycinins consist of six subunits linked by disulfide bonds, but the five major subunits are G1–G5 whose molecular weights range from 54 to 64 kDa. Prolamins are the major proteins in endosperm and they are more variable than globulins. In maize grain, zeins are the most abundant storage proteins and are mainly accumulated in the endosperm between 12 and 40 days after pollination [96]. They are grouped into α (19 and 22 kDa), β (16 kDa), γ (16, 27 and 50 kDa) and δ (10 kDa) zeins [63]. Wheat (*Triticum aestivum* L.) prolamins, gliadins and glutenins form gluten and are located in grain endosperm. Gliadins are often subdivided into various subtypes in accordance with their electrophoretic mobilities (i.e., α-, β-, γ- and ω-gliadins), whereas glutenin subunits are subdivided according to their molecular weights (i.e., HMW-GS and LMW-GS glutenins). In rice (*Oryza sativa* L.), glutelins are the major seed storage proteins that contain hexamers of α-polypeptides (35 kDa) and β-polypeptides (22 kDa). Storage proteins are abundant proteins but subtypes are differentially represented in seed/tuber proteomes with relative amounts ranging from 1–4% (δ-zeins, *Z. mays*) to 72% (7S lentil vicilins, *Lens culinaris* Medik.), whereas *M*_r_-values range from 10 (δ-zeins, *Z. mays*) to 130 kDa (HMW-GS glutenins, *T. aestivum*) (Table 1).

## 3. Two-Dimensional-Based Reference Maps of Storage Proteins

2-DE can be routinely applied for the separation of highly complex mixtures of proteins from cell, tissue, organ and organism protein extracts in accordance with their isoelectric point (*pI*) and molecular mass (*M_r_*) in two successive steps: isoelectric focusing (IEF) in the first dimension and sodium dodecyl sulphate-polyacrylamide gel electrophoresis (SDS-PAGE) to resolve denatured proteins in the second. The introduction of immobilized pH gradients (IPGs) using bifunctional immobiline reagents enabled us to obtain highly stable pH gradients in the first dimension increasing resolution, reproducibility, the detection of lower abundance proteins and the separation of highly acidic and alkaline proteins [97,98]. Many other technical achievements contributed to the optimization of 2-DE, such as more efficient protein extraction methods, the running of multiple gels in parallel, highly sensitive protein stain methods based on fluorescent dyes compatible with subsequent protein identification by MS technologies, and advanced computer software for the analysis of gel images [99,100,101,102,103]. Technical inter-gel variation of protein spots can be reduced using an internal pooled standard in multiplexing methods. Difference gel electrophoresis (DIGE) enables the simultaneous running of up to three different samples in a single 2-DE gel using pre-electrophoretic labeling of protein samples with distinct spectrally-resolvable fluorescent CyDyes [101].

Dedicated protein extraction protocols can alleviate in part some of the limitations of the standard 2-DE system, including the analysis of low-abundant proteins and membrane proteins [98,102,104,105]. It is noteworthy that plant tissues contain relatively lower amounts of proteins than other organisms and a large number of biological compounds that interfere notably with the extraction, solubilization and separation of proteins by 2-DE, such as cell walls, lipids, polysaccharides, polyphenols and large quantities of proteases. Therefore, protein extraction is the initial and one of the most critical steps in plant proteomic studies because it determines to a large extent the final quality of 2-DE [99,106,107,108,109]. Overall, 2-DE is a laborious and poorly automated technology that requires a great deal of expertise to successfully exploit its potential.

High-resolution 2-DE can successfully separate, detect and quantify up to thousands of proteins simultaneously [99]. It is routinely applied in current proteomics to effectively analyze abundant and soluble proteins with an amount of 1–2 ng per spot expressed at greater than 10^3^ copies/cell, a linear dynamic range about three orders of magnitude, molecular mass ranging from 15 to 150 kDa and pH intervals from 2.5 to 12 [99,104,110]. Accordingly, 2-DE has enough resolving power to separate most of the isoforms of storage proteins. These proteins are soluble and highly abundant, exhibiting a range of *M*_r_ and p*I* within 2-DE resolution limits. By way of illustration, values of *M*_r_ over phaseolin and patatin isoforms have a range of variation between 40 and 50 kDa, whereas p*I*-values range from 4.5 to 5.8 [29,30,42,44]. Gel location of storage protein isoforms can be initially established in accordance with their theoretical *M*_r_ and p*I* values and candidate protein spots eventually confirmed by MS for polypeptide/protein identification. 2-DE has the important ability to detect degraded proteins by comparing their *M*_r_ values observed on gels to those corresponding theoretical values [109,111].

**Figure 1:**
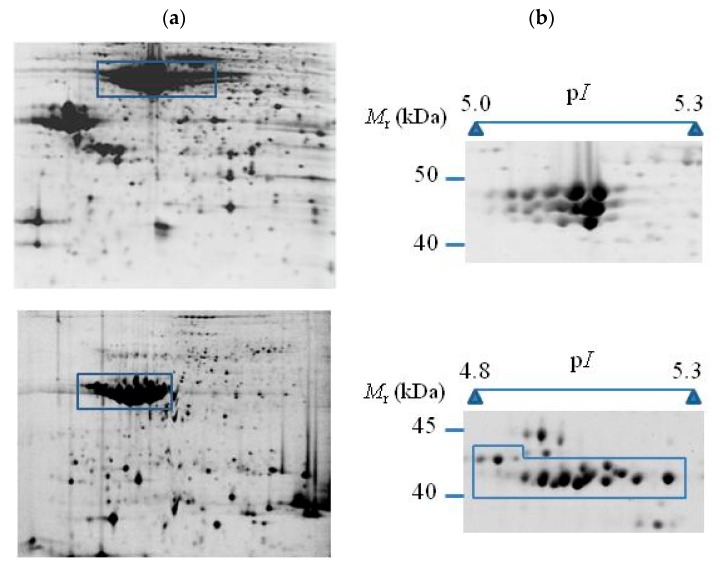
Standard (**a**) and targeted (**b**) 2-DE gel images of phaseolin (above) and patatin (below) isoforms from common bean (*P. vulgaris*) seeds and potato (*S. tuberosum*) tubers. Standard 2-DE gels were obtained from 250 μg of total seed protein or total tuber protein extracts loaded into 24-cm-long IPG strips of linear pH gradient 4–7 in the first dimension. The second dimension (SDS-PAGE) was run on 12% (*w*/*v*) SDS-PAGE gels. Gels were subsequently stained with SYPRO Ruby fluorescent stain. Targeted 2-DE gel images for high-resolution profiles were obtained under the same conditions but using only 75 μg of total protein extracts.

**Figure 2:**
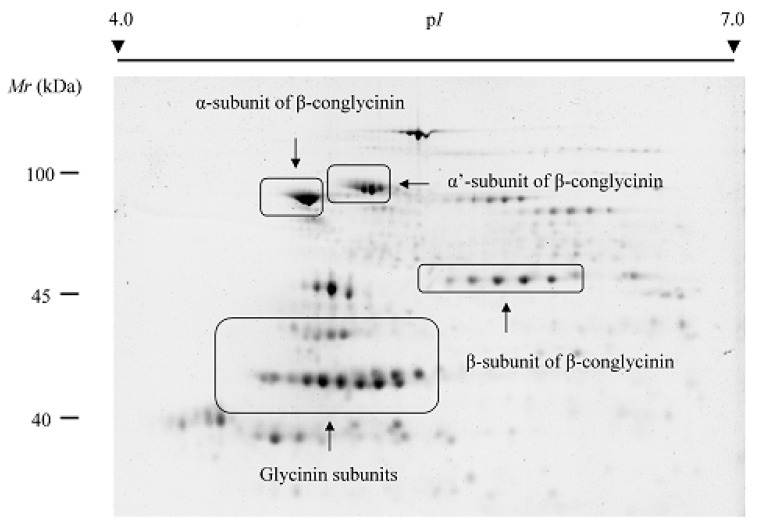
Gel image of high-resolution profile of soybean (*G*. *max*) storage proteins (β-conglycinin and glycinin subunits) obtained by the targeted 2-DE.

High-resolution profiles for storage proteins can be achieved by conveniently adjusting the amount of total protein loaded onto IPG strips [29,30,42,43,44]. Figure 1 shows standard and optimized phaseolin and patatin profiles by loading low amounts of total protein extracts from common bean seeds and potato tubers, respectively. It can be seen that dedicated 2-DE protocols produce good quality gel images with well-focused and separate protein spots corresponding to different phaseolin and patatin isoforms. 2-DE phaseolin and patatin profiles comprise a large number of spots organized in a compact way on the same gel region. Protein storage profiles can also exhibit multiple constellations of spots widely distributed on 2-DE gels (Figure 2). Dedicated 2-DE protocols have the additional advantage that the statistical cost by probability adjustments for multiple hypothesis testing is lower than in protocols addressed to the analysis of total seed proteomes, which leads to an increase in the statistical power of significance tests.

**Figure 3:**
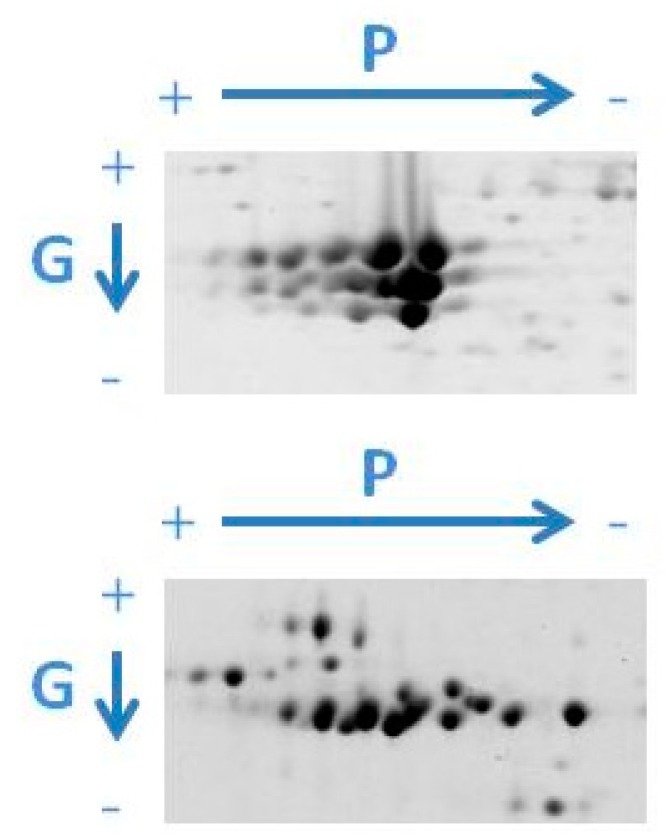
Gel images of differentially phosphorylated (P) and glycosylated (G) isoforms of phaseolin (above) and patatin (below) obtained by targeted 2-DE.

2-DE is particularly useful for identification of PTMs that change the p*I* and/or *M*_r_ of proteins such as phosphorylations and glycosylations [102,112]. 2-DE-based reference maps of storage proteins can, therefore, be implemented with in-gel detection and mapping of phosphorylated and glycosylated isoforms (Figure 3). The Pro-Q diamond phosphoprotein stain (Pro-Q DPS) is a simple, direct, rapid and commonly used method for in-gel multiplex detection, mapping and quantitation of phosphorylated proteins [113,114]. Recent studies indicate, however, that the phosphoprotein chemical dephosphorylation of seed protein extracts with hydrogen fluoride-pyridine (HF-P) [115] prior to 2-DE is a highly valuable strategy for more accurate in-gel quantitation of phosphorylated storage proteins [29,30]. Phosphorylation levels for 2-DE spots can be directly assessed from volume changes between dephosphorylated and control sample profiles.

The analysis of phosphorylated isoforms of storage proteins based on dedicated 2-DE maps has several major advantages in comparison to MS-driven analyses. A standard “bottom up” quantitative phosphoproteomics workflow involves the enzymatic or chemical digestion of a mixture of proteins into peptides to produce MS/MS spectra [33]. The redundancy of peptides and phosphorylation sites over high sequence identity protein isoforms hinders the assignation of specific peptides to a single isoform [112]. It is noteworthy that storage protein isoforms are encoded by gene families that exhibit high sequence identity mainly due to concerted evolution mechanisms of unequal crossing over and gene conversion [116]. For instance, patatin isoforms are encoded by a multigene family constituted by ~10–18 genes per haploid genome [28] and exhibit a sequence homology of at least 90% [58,117]. In addition, many other factors can lead to erroneous conclusions in MS-driven PTM analysis such as the co-elution of peptides, the loss of phosphoryl group during ionization process and phosphate transfer to acceptor residues, a lack of reproducibility and a low number of commonly used biological replicates [96,112,118]. It is noteworthy that some of these methodological constraints apply to powerful MS-based methods used for quantitative proteomics such as stable isotope labeling with amino acids in cell culture (SILAC) and isobaric tags for relative and absolute quantitation (iTRAQ). On the other hand, phosphopeptide enrichment strategies are usually accomplished prior to MS analysis because of the fact that many phosphoproteins/phosphopeptides from biological samples may be present in substoichiometric amounts [33,118]. In the case of storage proteins, the application of enrichment methods is not required because they are abundantly phosphorylated proteins [29,30]. Phosphopeptide enrichment methods such as immobilized metal affinity chromatography (IMAC) and titanium dioxide (TiO_2_) impair the evaluation of quantitative changes in the phosphorylation status among storage protein isoforms, although they are very useful for phosphosite identification.

Finally, the detection and quantitation of glycosylated isoforms of storage protein can be assessed by different methods, including the enzymatic deglycosylation of total protein extracts [42], in-gel glycoprotein-specific Pro-Q Emerald fluorescent stain [119] and glycopeptide enrichment using a zwitterionic (ZIC) hydrophilic interaction liquid chromatography (HILIC) column or affinity chromatography on a concanavalin-A-sepharose column [120,121]. Storage protein glycoforms can be identified efficiently by their *M*_r_ shifts on gels using targeted 2-DE protocols [30,42]. Glycosylated peptides are often difficult to identify in MS analyses because glycosylations change the hydrophobicity/hydrophilicity of the peptide [110].

## 4. Advances in the Biology of Storage Proteins

An exhaustive number of studies using 2-DE-based maps have contributed significantly to the characterization of the wide diversity of types, subunits and isoforms of storage proteins, their relative abundance in seeds and tubers, PTMs, targeted mutation effects and both qualitative and quantitative variations within and between wild and cultivated accessions [29,30,31,32,33,34,35,39,40,41,42,43,44]. In addition, 2-DE-based maps have provided valuable information on the complex dynamic changes of storage proteins during seed development and germination.

### 4.1. Seed Development

The available evidence indicates that storage proteins accumulate following variable patterns during embryo growth and seed filling, depending on the type of storage protein and cultivar. Thus, Gallardo et al. [4] reported that the major storage proteins 11S legumins and 7S vicilins of the model legume *Medicago truncatula* L. are synthesized in a specific temporal order and accumulated in different relative amounts during seed development. Analysis of protein abundance changes during time course were assessed by 2-DE and protein identification by MALDI-TOF and nano-LC-MS/MS sequencing. Interestingly enough, they also found a parallel evolution in the expression of the *pII* gene involved in the regulation of the synthesis of the amino acid arginine needed for storage protein synthesis using a transcriptomics dataset. Guo et al. [67] reported that five types of wheat storage proteins (i.e., γ-gliadins, globulins, avenin-like proteins, triticins and LMW-S glutenin subunits) accumulated differentially during grain development using 2-DE and tandem MALDI-TOF/TOF MS. This study also showed that LMW-S glutelin subunits and triticins exhibited differential abundance in two Chinese bread wheat cultivars at late seed development stages. In contrast, storage proteins of rapeseed (*Brassica napus* L.), i.e., napins, cruciferins and oleosins, were found to be accumulated only during the early and middle stages of seed growth by applying histochemical and inmunostaining techniques [20,122]. In addition, recent studies have revealed that different isoforms of phaseolin/patatin are differentially accumulated during seed/tuber development within and among cultivars from quantitative analysis of phaseolin/patatin isoforms using dedicated 2-DE protocols and protein identification by MALDI-TOF/TOF MS [29,30,42,43,44]. Taken together, these observations raise the question of the molecular and biochemical mechanisms responsible for differential accumulation of storage protein isoforms during seed/tuber development, but they also suggest that the differential accumulation of storage proteins and isoforms has a significant meaning for their mobilization in the germination stage.

Reversible phosphorylation is the most ubiquitous and well-studied type of PTM that regulates a huge variety of key biological processes, including cell cycle, metabolism, subcellular locatization, apoptosis, and signal transduction pathways [33,123,124]. The analysis of temporal phosphorylation changes in storage proteins is of paramount importance to unraveling their functional role at different stages of seed development. In recent decades the number of reports on the phosphorylation of different SSPs during seed development, dormancy and germination has greatly increased: globulins (7S- and 11S-globulins, 12S cruciferin, 12S triticin, cupin and globulin 3) in Arabidoposis, common bean, rapeseed, rice, Scots pine (*Pinus sylvestris* L.), sunflower (*Helianthus annuus* L.) and wheat [29,67,83,125,126,127,128,129,130,131,132]; prolamins in wheat [133,134]; albumins (2S napin) in Scots pine and Arabidopsis [126,127,130]; and glutelins in rice [135]. Bernal et al. [30] have recently reported the first evidence for the phosphorylation of VSPs in patatin.

Identification and profiling studies of phosphorylated storage proteins based on 2-DE maps combined with various other techniques are listed in Table 2. Phosphoproteomic studies show that storage proteins are abundantly phosphorylated and may play a key role during seed development. Meyer et al. [129] reported a large-scale MS-based study of enriched subproteome of phosphoproteins by the IMAC method at five sequential stages (2–6 weeks after flowering) of seed development in soybean, rapeseed and Arabidopsis. A total of 2001 phosphopeptides and 1026 unambiguous phosphorylation sites were identified across 956 non-redundant proteins, including storage proteins. Interestingly, a considerable fraction (25%) of phosphoproteins consisted of storage proteins that contained the X-S-D-X phosphorylation motif. Targeted 2-DE-based maps coupled to the chemical method of dephosphorylation with HF-P have shown high phosphorylation levels in storage protein isoforms. Phosphorylation rates over phaseolin isoforms in dormant common bean seeds (two cultivars) and patatin isoforms from mature potato tubers (one cultivar) measured by the *PR* coefficient averaged 46–63% and 34%, respectively [29,30]. Furthermore, in silico phosphopeptide analysis also revealed the occurrence of a putative phosphosite in phaseolin phosphopeptides encompassing sequence X-S-D-X in the phaseolin. This peptide, therefore, appears to be a general target for phosphorylation during seed development.

2-DE-based maps show that the accumulation of phosphorylated storage protein isoforms during seed filling also follows variable patterns. Agrawal and Thelen [125] performed the first comprehensive study aimed at detecting and quantifying phosphoproteins in development seeds. More specifically, phosphoprotein profiling was performed in rapeseed through the same five sequential phases of seed development as Meyer et al. [129] by means of 2-DE-based maps coupled to in-gel phosphoprotein specific staining with Pro-Q DPS fluorescent dye and LC-MS/MS for protein and phosphorylation site identification. The results of the study showed that 40% of phosphorylated cruciferin subunits increased during seed filling process, whereas the remaining phosphorylated subunits generally decreased with seed development. Meyer et al. [129] also reported that some phosphorylated cruciferin subunits were over-represented in the late maturation stage of seed development. Dedicated 2-DE protocols have disclosed that phosphorylation rates (PR) across different phaseolin/patatin isoforms from dormant seed/tuber were in the range of 13–82% and 5–52%, respectively [29,30].

The complex regulatory mechanisms underlying dynamic changes in the phosphorylation status of storage proteins in response to seed development and environmental factors are not yet sufficiently known. However, it is assumed that the interplay of protein kinases, protein phosphatases and phytohormones participates in the signaling and metabolic networks that control the phosphorylation/dephosphorylation levels of storage proteins. The CK2 protein is a Ser/Thr kinase presents in all eukaryotes and has pleiotropic effects; it is also involved in the regulation of multiple plant growth and development processes and ABA signalling [136,137,138]. Irar et al. [126] used 2-DE-based maps for the phosphoproteome profiling of heat-stable proteins from Arabidopsis dry seeds and phosphoaffinity chromatography for phosphoprotein enrichment. They reported several probable hits of phosphorylation in storage and like-storage proteins, and an increased probability of phosphorylation of serine over threonine residues by CK2, using in silico prediction of phosphorylation sites from MALDI-TOF MS and LC MS/MS data. On the other hand, the ABA-insensitive 1 (ABI1) protein phosphatase is a negative regulator of the ABA signal and interacts with proteins linked to the ubiquitin-proteosome system (UPS) [139,140]. Wan et al. [127] showed that cruciferins of *A. thaliana* may be an in vivo target for ABI1 during seed development and provided evidence that cruciferin phosphorylation levels might be regulated by ABI1 using 2-DE maps coupled to immunological detection against phosphorylated cruciferin. They also found that cruciferins had differential levels of Tyr phosphorylation in mutant *ABI1* and wild types, which suggests that Tyr phosphorylation is involved in ABA signaling.

### 4.2. Seed Germination

2-DE-based proteomic analyses revealed that the accumulation of storage proteins can still proceed in late stages of seed development and the onset of germination. Chibani et al. [141] reported that cruciferin precursors in Arabidopsis are accumulated by de novo synthesis during late stages of seed development leading to dormancy breakage. The accumulation of cruciferin precursors was documented by 2-DE following protein identification by MALDI-TOF MS. Proteomic research on Arabidopsis seed dormancy by 2-DE coupled to MALDI-TOF MS from seeds of the GA-deficient ga1 mutant and wild-type seeds treated with a specific inhibitor of GA biosynthesis suggests that GA is involved in the processing of precursor forms of storage proteins and accumulation of processed forms in mature seeds [142]. The comparison of 2-DE patatin profiles in dormant tubers and the onset of germination led to a better understanding of the metabolic status of storage proteins after the dormancy break. Lehesranta et al. [143] reported temporal differences of patatin abundance throughout the potato tuber lifecycle (cv. Desirée). More specifically, it was found that most patatin isoforms increase during development, are present in high amounts at the onset of sprouting (i.e., sprouts ca. 1 cm long) and remain approximately constant until tubers are fully sprouted (i.e., sprouts ca. 20 cm long) when patatin abundance decreases. Accordingly, analyses on transcripts encoding patatin throughout the potato tuber cycle based on cDNA-AFLP fingerprinting and expressed sequence tag (EST) libraries have shown that patatin transcripts are still expressed at the onset of tuber sprouting [144,145]. Similar results have been reported after chemically (bromoethane) induced cessation of dormancy using microarrays constructed from potato EST libraries [146]. Overall, these studies suggest that the major tuber storage protein encoded by the patatin multigene family is also synthesized after the dormancy break to ensure growth of the developing sprout.

Changes in the abundance or phosphorylation status of storage proteins during seed germination have been monitored using 2-DE-based reference maps [29,128,131,132]. Ghelis et al. [128] reported that the status of Tyr phosphorylation for several cruciferin precursors and cruciferin subunits in Arabidopsis seeds was modulated in response to ABA using 2-DE-based maps and the identification of phosphorylated Tyr residues by means of anti-phosphotyrosine antibodies in western blots. It was found that cruciferins treated with ABA exhibited higher phosphorylation levels than control seeds. In rice, Han et al. [131] detected that the highest level of phosphorylation of cupins coincided with the late stage of germination and protein degradation by means of 2-DE combined with Pro-Q DPS staining and MALDI-TOF/TOF MS. Using DIGE-based maps, Dong et al. [132] detected an increased abundance of phosphorylated wheat globulin 3 at 12 h after imbibition. In common beans, the analysis of targeted 2-DE-based phaseolin profiles coupled to protein dephosphorylation with HF-P revealed changes in the phosphorylation status during dry-to-germinating seed transition [29]. Changes in the phosphorylation status unexplained by parallel variations in the amount of protein are suggestive of their functional role [96]. Importantly, highly phosphorylated phaseolin isoforms were preferentially degraded in germinating seeds. These results support the conclusion that phosphorylation-dependent degradation plays a significant role in the mobilization of phaseolin. It has been suggested that phosphorylation can cause conformational changes in the protein and promote its mobilization during germination [127]. Overall, the molecular pathways, phosphorylation sites and specific kinases/phosphatases governing variations in phosphorylation status are totally unknown.

Protein glycosylation is involved in the modulation of relevant biological processes such as protein folding, protein stability, protein-protein interactions and interaction with membrane components [147,148,149]. Asparagine (N)-linked glycosylation is the major co- and post-translational modification of proteins in plants [150]. The application of a great diversity of molecular techniques permitted the identification of glycosylated isoforms in many types of storage proteins and species: globulins (7S- and 11S-globulins and convicilin) in adzuki bean (*Vigna angularis* L.), blue lupins (*Lupinus angustifolius* L.), cocoa beans (*Theobroma cacao* L.), common beans, hazelnuts (*Corylus avellane* L.), lentils, Lotus (*Lotus japonicus* L.), mung beans (*Vigna radiata* L.), peas (*Pisum sativum* L.), peanuts (*Arachis hipogea* L.), soybeans and white lupin (*Lupinus albus* L.) [42,120,121,151,152,153,154,155,156,157,158,159,160,161,162,163,164,165,166,167,168,169,170,171,172,173,174,175,176,177,178,179]; prolamins (γ3-hordein) in barley [180]; albumins (3S albumins) in Inca peanuts (*Plukenetia volubilis* L.) [181]; glutelins in rice [135,182]; VSPs (patatin) in potatoes [30,43,183,184,185,186]; and lectins (monocot mannose-binding lectin, phytohemagglutinin) in air potatoes (*Dioscorea bulbifera* L.), common beans and lotus [121,187,188,189,190,191,192,193].

Identification and profiling studies of glycosylated storage proteins using 2-DE-based maps together with various other techniques are listed in Table 3. Most of these studies are addressed to the identification of glycosylated isoforms, the assessment of differential degrees of glycosylation and effects in food allergy. The biological role of glycosylated forms remains largely unknown. Interestingly, Santos et al. [177] reported that the glucoside hydrolase β-*N*-acetylhexosaminidase (β-NAHase) is involved in α-conglutin mobilization in white lupin storage proteins.

## 5. Application Areas in Seed Breeding

### 5.1. Seed Quality

Seed protein quality is an essential trait in seed breeding programs. The nutritional quality of proteins is largely dependent on their essential amino acid (EAA) composition, total protein content and digestibility. Seed proteins are often deficient in specific EAA such as lysine, tryptophan, threonine and methionine. For example, high relative concentrations of lysine can be found in potato tuber but it is a nutritionally limiting EAA in most cereals [194]; whereas soybeans and common beans are deficient in methionine [194,195]. Storage proteins are abundant and determine to a great extent seed protein quality. For example, the relative abundance of prolamins in cereals has a key influence for protein quality because of their deficiences in EAA [73]. In particular, zein is a prolamin that accounts for between 50 and 70% of the total seed protein of maize and is mainly deficient in the content of lysine and tryptophan followed by methionine [61,196,197]. The particular mix of abundant storage proteins can also determine the final quality of seed proteins. For example, glycinin (11S legumin type) and conglycinin (7S vicilin type) are the two major soybean storage proteins, but glycinin harbors three to four times more sulfur-containing amino acids than conglycinin [198].

2-DE-based maps are a very effective tool for screening and selecting varieties containing specific protein storage isoforms linked to high protein quality in plant breeding. This proteomic approach has been addressed in a variety of crops. For instance, wild rice species are a valuable source of genetic resources for improving the nutritional quality of rice by increasing the glutelin content to the detriment of prolamins [199,200]. The comparison of 2-DE-based maps between wild rice species and rice cultivars revealed new subunits and precursors of glutelin in wild rice species [199]. 2-DE gels also revealed that the content of glutelins in an ancient Chinese wild rice (*Zizania latifolia* (Griseb.) *Turcz.*) was approximately twice as high as that of the Indica rice cultivar [200]. Zarkadas et al. [195] also reported great variability among soybean cultivars for glycinin and β-conglycinin using 2-DE. In common bean, López-Pedrouso et al. [44] reported that pairwise proteomic distances estimated from wild and domesticated accessions of the major Mesoamerican and Andean gene pools assessed by targeted 2-DE of the phaseolin provide valuable information for identifying outlier cultivars with increased content in methionine.

A number of factors modeling the genetic structure of populations can generate and/or maintain genome-wide non-random associations between alleles at different loci (linkage or gametic disequilibrium) such as founder effects, bottlenecks, inbreeding and selection [201]. These factors or combination of factors often operate in plant breeding. Accordingly, storage proteins encoded by multigene families can be used to detect nonrandom associated quantitative trait loci (QTLs) underlying quality traits. In this regard, the nutritional quality of protein and the starch content and average weight of potato tubers were found to be correlated with patatin content [202,203].

Different types of transgenic-based strategies have been addressed at the improvement of seed protein quality from storage proteins. Some strategies rely on the ectopic expression of transgenes coding for high quality proteins that correct seed deficiencies in the amino acid composition of storage proteins. Shekhar et al. [50] introduced the seed albumin gene *AmA1* from *Amaranthus hypochondriacus* into sweet potato (*Ipomoea batatas* L.) by Agrobacterium-mediated transformation to assess the behavior of storage proteins in a non-native system. *AmA1* is rich in all EAA whereas sweet potato proteins are deficient in tryptophan and sulfur-containing amino acids. Comparative proteomics revealed that 2-DE profiles of transgenic tubers exhibited a higher number of protein spots than wild-type tubers. The results suggest that overexpression of *AmA1* in sweet potato tubers seems to have a marked effect on nutrient acquisition, which facilitates an increase in the overall protein and amino acid content. Other alternative transgenics-based approaches are used to overproduce one particular seed protein with higher nutritional quality than the remaining set of storage proteins. For example, the overexpression of glycinin enables an increase in sulfur amino acids in soybean seeds, taking into account that the content of glycinin correlates negatively to the content of β-conglycinin [204]. El-Shemy et al. [198] transformed soybean embryos with a chimeric proglycining gene encoding a methionine-rich glycinin. The comparison of transgenic and untransformed soybean lines by 2-DE revealed an increased accumulation of glycinin in transgenic soybeans.

### 5.2. Gluten Disorders and Allergies

Gluten proteins and gluten-like proteins are the main factor triggering coeliac disease (CD), non-coeliac gluten sensitivity and gluten allergies in genetically susceptible individuals [51,205,206]. CD is an autoimmune condition caused by human intolerance to wheat gluten and related proteins from rye (secalins, *Secale cereale* L.), barley (hordeins) and oat (avenins, *Avena sativa* L.) that primarily affect the small intestine [72,206]. Gluten is composed of a combination of two toxic prolamines in CD, glutenins and gliadins, but gliadins contain most of the epitopes triggering CD [51,72]. A gluten-free diet is often low in fiber and minerals, high in sucrose and saturated fatty acids, and more expensive [207,208]. A wide-variety of strategies have been applied for the selection and breeding of less toxic varieties. These include obtaining varieties with a lower dose or a different composition of gluten proteins. García-Molina et al. [51] carried out a 2-DE-based proteomic study to evaluate the effects of the strong down-regulation of gliadins on the expression of target and non-target proteins. For this purpose, transgenic wheat lines with downregulation of gliadin expression were obtained by RNA interference (RNAi) technology. As expected, transgenic lines showed a lower abundance of gliadins with respect to control lines. However, the glutelin fraction and other allergen-related wheat proteins increased in low-gliadin lines by a compensation effect. Kawaura et al. [209] obtained aneuploid wheat lines to reduce CD immunotoxicity in breeding programs. An analysis of 2-DE profiles disclosed that α-gliadins containing major CD epitopes were lost in tetrasomic lines. In barley, Tanner et al. [206] obtained an ultra-low gluten variety (hordein content below 5 ppm) by combining three recessive alleles with potential application in the preparation of foods and beverages for CD patients and people who cannot tolerate gluten. Only reduced amounts of the γ-3-hordein protein were observed in the ultra-low gluten variety by 2-DE, in accordance with other protein quantitative determinations. 2-DE also contributed to demonstrating that wheat α-gliadins can be compensated by the addition of avenins to the floor to improve dough quality, taking into account that a minority of CD patients are sensitive to oat avenins [210]. Rizzello et al. [211] showed by in vitro analysis that making bread from flour with an intermediate content of gluten improves its digestibility and nutritional quality without the loss of the chemical, structural and sensory characteristics of traditional breads. 2-DE revealed increased protein degradation in flour with an intermediate content of gluten during fermentation. The authors suggested that this wheat product might be useful to prevent, delay or treat susceptibility to gluten sensitivity, a gluten reaction that does not involve allergic or autoimmune mechanisms.

### 5.3. Seed Longevity

Dry seed longevity is an essential complex trait for the biodiversity conservation of cultivated plants. Seed longevity and the germination vigor rate slowly decrease during storage ageing, influenced by abiotic and biotic variables, including storage conditions (e.g., temperature and humidity) and genetic factors [212,213,214]. Compelling evidence indicates that antioxidant systems (antioxidative enzymes and antioxidants) deteriorate during seed ageing leading to the accumulation of reactive oxygen species (ROS) and oxidative damage [49,124,215]. SSPs undergo extensive oxidization (often carbonylation) during long-term seed storage due to their abundance and high affinity to oxidation [49,215,216,217]. Seed ageing profiling in rice assessed by 2-DE followed by western blotting with antidinitrophenyl hydrazone antibodies revealed that carbonylated SSPs accumulate at the critical node of seed ageing leading to a rapid decline in seed viability [124]. Nguyen et al. [49] proposed that SSPs may be buffers for seed oxidative stress, able to protect relevant proteins for seed germination and seedling development from proteomic profile analysis of Arabidopsis cruciferin mutants based on 2-DE and LC-MS/MS. Dobiesz et al. [214] reported that β- and δ-conglutins may be a useful biomarker of lupin (*Lupinus luteus* L.) seed viability during long-term storage using 2-DE and LC-MS/MS.

### 5.4. Other Applications

The analysis of storage proteins by 2-DE-based maps has also contributed to the development of other application areas such as antifungal, antibacterial and insect susceptibility [45,46,47,218], the identification of allergens [46], drought stress [48,219], wheat cultivar identification in blended flour [220] and the large-scale production of therapeutic proteins [221].

## 6. General Conclusions and Perspectives

This review shows that the use of 2-DE combined with MS is of vital importance not only to advancing the knowledge of the isoforms of storage proteins and their dynamic changes during seed development and germination in a wide diversity of plants, but also in relevant fields closely connected to seed breeding. Therefore, the employment of 2-DE is expected to follow over the next years due to its high efficiency in the characterization of storage proteins across different biological scenarios. Gel-based and shotgun proteomics are alternative strategies for proteome analysis that have advantages and limitations but complement each other. The joint use of gel-based and gel-free methodologies will probably continue to be necessary in follow-up studies to understand the complex biology of storage proteins. Despite significant progress over the last decades, proteomics faces major challenges in the coming years to unravel the complex molecular puzzle of regulatory networks underlying the activities, functions, and interactions of storage proteins over the lifecycle of seeds. In particular, further experiments are clearly needed to assess the exact role of phosphorylated isoforms and specific phosphorylation sites during seed development and germination. This huge task will probably require the integration of multi-omics data with the help of new bioinformatic tools.

## Figures and Tables

**Table 1:** List of seed and vegetative storage proteins in different crop types.

Crop	Storage Proteins	Percentage of Total Protein	Molecular Weight Subunits (kDa)	References
				
Maize (*Zea mays* L.)	Globulins	12–16		[3,60,61,62,63,64]
	globulin-1		63, 45, 26, 23	
	globulin-2			
	Prolamins	50–70		
	α-zeins	25–49	22, 19	
	β-zeins	1–4	14–16	
	γ-zeins	6–13	27, 16, 50	
	δ-zeins	1–4	10	
				
Wheat (*Triticum aestivum* L.)	Prolamins	80		[65,66,67,68,69,70,71,72,73]
	gliadins	30–50	30–80	
	α-gliadins	15–30		
	β-gliadins			
	γ-gliadins			
	ω-gliadins			
	glutenins			
	LMW-GS	12	42–51 (B), 30–40 (C), 58 (D)	
	HMW-GS		80–130 (A)	
	Globulins			
	11-12S triticins	5	58 (D), 22 (δ), 52 (A), 23 (α)	
				
Rice (*Oryza sativa* L.)	Glutelins	60–80	35–40, 20–22	[74,75,76]
	Prolamins	20–30	10, 13, 16	
	Globulins			
	α-globulins	2–8	26	
				
Potato (*Solanum tuberosum* L.)	Patatins	45	39–45	[30,77,78]
	Kunitz protease inhibitors		20	
	Protease inhibitors 1		45	
	Protease inhibitors 2			
	Carboxypeptidase inhibitors		10	
	Lipoxygenases		97	
				
Soybean (*Glycine max* L.)	Globulins			[79,80,81]
	α-conglycinins			
	7S vicilin/β-conglycinins	40	76 (α), 72 (α’), 52 (β)	
	γ-conglycinins			
	11S legumin/glycinins	25	56 (G1), 54 (G2), 54 (G3), 64 (G4), 58 (G5)	
				
Barley (*Hordeum vulgare* L.)	Prolamins			[68,82]
	hordeins	35–55		
	B-hordeins	15–44	30–45	
	C-hordeins	4–11	45–75	
	D-hordeins		45	
	γ-hordeins			
				
Sunflower (*Helianthus annuus* L.)	Globulins			[83,84,85]
	11S helianthinins	38	37–43 (α), 31–35 (α’), 21–30 (β)	
	Albumins			
	2S	62	12–20	
				
Common Bean (*Phaseolus vulgaris* L.)	Globulins			[14,44,86,87]
	7S phaseolins	40–50		
	11S legumins	3		
	Lectins			
	phytohemagglutinins	5–10		
	α-amylase inhibitors			
				
Oat (*Avena sativa* L.)	Globulins	10–55		[71,88,89]
	3S		48–52	
	7S		50–70	
	11S		60	
	12S avenalins		32–43 (α), 19–25 (β)	
	Albumins	10–20		
	Prolamins	12–14		
	Glutelins	23–54		
				
Pea (*Pisum sativum* L.)	Globulins			[90,91]
	7S vicilins		47, 50, 34, 30	
	11S legumins		41 (α), 22 (β), 23 (β’)	
	convincilins		78, 72	
				
Chickpea (*Cicer arietinum* L.)	Albumins			[89,92]
	2S	12		
	Globulins	50		
	7S vicilins			
	11S legumins		40–47 (α), 24–25 (β)	
	Glutelins	18.1		
	Prolamins	2.8		
				
Pomegranate (*Punica granatum* L.)	Globulins	40.5	38–54, 13–18	[93]
	Albumins	32.2	58–116, 33–46, 15–23	
	Glutelins	15.6	37, 21–23, 14	
	Prolamins	9.7	15, 20, 24	
				
Lentils (*Lens culinaris* Medik.)	Globulins			[94]
	11S legumins	21	38–43	
	7S vicilin/ convicilins	72	15–59	
	Albumins			
	2S			
				
Rapeseed (*Brassica napus* L)	Globulins			[95]
	12S cruciferins	20	29–33 (α), 21–23 (β)	
	Albumins			
	2S napins	60	4–9	

**Table 2:** List of 2-DE-based seed phosphoproteomic studies including storage proteins.

Storage Protein Type	Storage Protein Subtype	Seed Stage	Additional Techniques	Species	References
					
Globulin	12S cruciferin	Development	Pro-Q DPS	Rapeseed (*Brassica napus* L.)	[113]
			LC-MS/MS		
				
	12S triticin Globulin 3	Development	Pro-Q DPS	Wheat (*Triticum aestivum* L.)	[67]
			MALDI-TOF		
			MALDI-TOF/TOF		
				
	12S cruciferin	Dormancy	1-DE, Pro-Q DPS	*Arabidopsis thaliana* L.	[126,127]
			immunoblotting		
			LC-MS/MS		
				
	7S phaseolin	Dormancy/ Germination	Pro-Q DPS, HF-P	Common bean (*Phaseolus vulgaris* L.)	[29]
			MALDI-TOF		
			MALDI-TOF/TOF		
				
	12S cruciferin	Germination	Western blotting	*Arabidopsis thaliana* L.	[128]
			MALDI-TOF		
			MALDI-TOF/TOF		
				
	Cupin	Germination	Pro-Q DPS	Rice (*Oryza sativa* L.)	[131]
			MALDI-TOF/TOF		
				
	Globulin 3	Germination	Pro-Q DPS	Wheat (*Triticum aestivum* L.)	[132]
			LC-MS/MS		
					
Albumin	2S napin	Dormancy	1-DE, Pro-Q DPS	*Arabidopsis thaliana* L.	[126,127]
			immunoblotting		
			LC-MS/MS		
					
Glutelin	N/A	Development	Pro-Q DPS	Rice (*Oryza sativa* L.)	[135]
			LC-MS/MS		
					
Vegetative	Patatin	Dormancy	Pro-Q DPS, HF-P	Potato (*Solanum tuberosum* L.)	[30]
			MALDI-TOF		
			MALDI-TOF/TOF		

N/A, not available.

**Table 3:** List of 2-DE-based seed glycoproteomic studies including storage proteins.

Storage Protein Type	Storage Protein Subtype	Additional Techniques	Species	References
				
Globulin	7S vicilin	1-DE, Glycoprotein staining	Cocoa bean (*Theobroma cacao* L.)	[179]
			
	7S phaseolin	1-DE, Fluorography	Common bean (*Phaseolus vulgaris* L.)	[42,161,162]
		Radioactive labelling of sugars,		
		Concanavalin A binding		
		Immunoaffinity chromatography		
		*N*-deglycosylation		
			
	7S convicilin	*N*-deglycosylation	Lotus (*Lotus japonicus* L.)	[121]
				
Glutelin	N/A	Glycoprotein staining,	Rice (*Oryza sativa* L.)	[135]
		LC-MS/MS		
				
Lectin	N/A	*N*-deglycosylation	Lotus (*Lotus japonicus* L.)	[121]
				
Vegetative	Patatin	*N*-deglycosylation	Potato (*Solanum tuberosum* L.)	[30,43,184]
		MALDI-TOF, MALDI-TOF/TOF		

N/A, not available.

## Abbreviations

1-DE	One-dimensional electrophoresis
2-DE	Two-dimensional electrophoresis
ABA	Abscisic acid
CD	Coeliac disease
DIGE	Difference gel electrophoresis
EAA	Essential amino acid
GA	Gibberellic acid
HF-P	Hydrogen fluoride-pyridine
IMAC	Immobilized metal affinity chromatography
*M_r_*	Relative molecular mass
MS	Mass spectrometry
p*I*	Isoelectric point
*PR*	Phosphorylation rate
Pro-Q DPS	Pro-Q Diamond phosphoprotein stain
PTM	Post-translational modification
SSP	Seed storage protein
VSP	Vegetative storage protein
